# CoolMPS: evaluation of antibody labeling based massively parallel non-coding RNA sequencing

**DOI:** 10.1093/nar/gkaa1122

**Published:** 2020-12-08

**Authors:** Yongping Li, Tobias Fehlmann, Adam Borcherding, Snezana Drmanac, Sophie Liu, Laura Groeger, Chongjun Xu, Matthew Callow, Christian Villarosa, Alexander Jorjorian, Fabian Kern, Nadja Grammes, Eckart Meese, Hui Jiang, Radoje Drmanac, Nicole Ludwig, Andreas Keller

**Affiliations:** Chair for Clinical Bioinformatics, Saarland University, 66123 Saarbrücken, Germany; MGI, BGI-Shenzhen, Shenzhen 518083, China; Chair for Clinical Bioinformatics, Saarland University, 66123 Saarbrücken, Germany; Complete Genomics Incorporated, San Jose, CA 95134, USA; Complete Genomics Incorporated, San Jose, CA 95134, USA; Complete Genomics Incorporated, San Jose, CA 95134, USA; Department of Human Genetics, Saarland University, 66421 Homburg, Germany; MGI, BGI-Shenzhen, Shenzhen 518083, China; Complete Genomics Incorporated, San Jose, CA 95134, USA; BGI-Shenzhen, Shenzhen 518083, China; China National GeneBank, BGI-Shenzhen, Shenzhen 518120, China; Complete Genomics Incorporated, San Jose, CA 95134, USA; Complete Genomics Incorporated, San Jose, CA 95134, USA; Complete Genomics Incorporated, San Jose, CA 95134, USA; Chair for Clinical Bioinformatics, Saarland University, 66123 Saarbrücken, Germany; Chair for Clinical Bioinformatics, Saarland University, 66123 Saarbrücken, Germany; Department of Human Genetics, Saarland University, 66421 Homburg, Germany; MGI, BGI-Shenzhen, Shenzhen 518083, China; MGI, BGI-Shenzhen, Shenzhen 518083, China; Complete Genomics Incorporated, San Jose, CA 95134, USA; BGI-Shenzhen, Shenzhen 518083, China; China National GeneBank, BGI-Shenzhen, Shenzhen 518120, China; Department of Human Genetics, Saarland University, 66421 Homburg, Germany; Chair for Clinical Bioinformatics, Saarland University, 66123 Saarbrücken, Germany; Department of Neurology and Neurological Sciences, Stanford UniversitySchool of Medicine, Stanford, CA 94304, USA

## Abstract

Results of massive parallel sequencing-by-synthesis vary depending on the sequencing approach. CoolMPS™ is a new sequencing chemistry that incorporates bases by labeled antibodies. To evaluate the performance, we sequenced 240 human non-coding RNA samples (dementia patients and controls) with and without CoolMPS. The Q30 value as indicator of the per base sequencing quality increased from 91.8 to 94%. The higher quality was reached across the whole read length. Likewise, the percentage of reads mapping to the human genome increased from 84.9 to 86.2%. For both technologies, we computed similar distributions between different RNA classes (miRNA, piRNA, tRNA, snoRNA and yRNA) and within the classes. While standard sequencing-by-synthesis allowed to recover more annotated miRNAs, CoolMPS yielded more novel miRNAs. The correlation between the two methods was 0.97. Evaluating the diagnostic performance, we observed lower minimal *P*-values for CoolMPS (adjusted *P*-value of 0.0006 versus 0.0004) and larger effect sizes (Cohen's d of 0.878 versus 0.9). Validating 19 miRNAs resulted in a correlation of 0.852 between CoolMPS and reverse transcriptase-quantitative polymerase chain reaction. Comparison to data generated with Illumina technology confirmed a known shift in the overall RNA composition. With CoolMPS we evaluated a novel sequencing-by-synthesis technology showing high performance for the analysis of non-coding RNAs.

## INTRODUCTION

Since the mid 1990′s, massively parallel sequencing approaches have been developed and continuously improved. The first commercial instruments were available on the market around 2005 (1). The rapid development of technology in the first 10 years had a substantial impact on genomic research (2), also leading to a continuous growth of data deposited in resources such as GenBank (3). While one of the most common applications is genome sequencing, RNAs are often analyzed using high-throughput sequencing as well. Even resolution at the single cell level can be reached now (4). A general overview of the different sequencing approaches together with available instruments highlights the diversity of available platforms and applications (5). Most recently, a comparison of Illumina NextSeq 500, NovaSeq 6000 and the BGI MGISEQ-2000 using identical single Cell 3′ libraries generated with the 10× Genomics Chromium platform highlighted comparable performance between the platforms in general (6).

For the high-throughput analyses of small non-coding RNAs (sncRNAs), sequencing has become one of the most frequently used methods (7). This has led to a very deep understanding of the sncRNA expression in humans (8,9) and many other species (10). As a consequence, databases on sncRNAs, especially on microRNAs (miRNAs) are updated regularly with increasing numbers of miRNAs. The miRBase in its most recent release 22 (October 2018 (11)) contains 38 589 entries from 271 species (12). Besides miRBase, MirGeneDB contains 10 899 curated miRNAs from 45 different organisms (13) and miRCarta (14) has the ambition to provide a collection of all expressed small RNAs. With 11 000 annual publications on miRNAs, these databases cover particular needs of researchers and provide an important source of information for future miRNA annotations (15). The largest fraction of miRNAs from high-throughput sequencing has been annotated for *Homo sapiens*. For example, as of August 2020, the miRMaster web service (16) has been applied in over 1300 studies. Sequencing data of more than 74 000 human sncRNA samples were evaluated and 1.1 trillion reads (1.1 × 10^12^) have been processed using miRMaster. Notably, only a fraction of all available sncRNA sequencing data has been analyzed using the miRMaster tool, e.g. since only one organism is considered. Thus, the total number of sncRNA sequencing data sets exceeds the figures given above substantially. The gold standard sncRNA analysis software miRDeep/miRDeep2 (17,18) for example has been cited almost 2000 times. Constantly decreasing cost and broader availability of sequencing systems will lead to a continuously growing amount of sncRNA datasets in the future.

Many studies, however, indicate a severe influence of sample handling, library preparation and the sequencing technology on the read quantity, composition and quality (19–22). The most commonly applied approach is sequencing-by-synthesis using Illumina systems. These are available in combination with different library preparation approaches (19). We previously evaluated the performance of sequencing-by-synthesis on Illumina systems to combinatorial probe-anchor synthesis (cPAS)-based BGISEQ-500 sequencer (23). As compared to the Illumina system we found a larger variety of sncRNAs in the cPAS data, including twice as much yet unknown microRNAs at that time. Both sequencing approaches however rely on similar sequencing-by-synthesis principles, incorporating labeled nucleotides during each sequencing cycle.

The continuous development of library preparation and sequencing approaches is leading to novel commercially available systems and assay formats. The availability of a new experimental approach however immediately calls questions with respect to the validity of its data and the comparability. Especially for applications in biomarker development a platform change may significantly affect the diagnostic or prognostic performance of tests. Consequently, two questions come up whenever a new experimental approach is available: how does the performance change if technical replicates are compared between platforms and how does it affect biological results?

Recently, a fundamentally novel sequencing approach called CoolMPS has been introduced and made commercially available through MGI Tech Co., Ltd, Shenzhen, China (details are provided in the ‘Materials and Methods’ section). While it still relies on the sequencing-by-synthesis principle as other methods, no labeled nucleotides are incorporated. In order to measure a signal intensity representative for the incorporated base at each cycle, four specific antibodies, one recognizing each of the four natural bases (A, T, C, G) are used. The approach promises higher data quality by avoiding incorporation and detection interference of base-linked dyes and providing stronger signals by attaching multiple molecules of a dye per antibody. The CoolMPS approach for sequencing non-coding RNAs is described in the ‘Materials and Methods’ section. More details on the sequencing kits and basic biochemical principles of the methodology and its application are available with the user manual of the commercial kits and as preprint (https://doi.org/10.1101/2020.02.19.953307). It is mandatory to evaluate such new technologies with respect to common application scenarios. Discovering single nucleotide variants or small insertions and deletions pose different challenges as compared to, e.g. the quantification of RNAs in an at least pseudo-quantitative manner. In this study, we set to present the first detailed and direct performance comparison between the novel antibody-based labeling approach in comparison to standard sequencing-by-synthesis using labeled nucleotides for the quantification of small non-coding RNAs.

## MATERIALS AND METHODS

### RNA sample preparation and quality control

RNA from 2.5 ml whole blood collected in PAXgene tubes was isolated using the PAXgene Blood miRNA Kit (Qiagen, Hilden, Germany) according to the manufacturer's recommendations. RNA concentration and integrity were measured using Nanodrop 2000 spectrophotometer (Thermo Fisher Scientific, Waltham, MA, USA) and RNA 6000 Nano Kit for Agilent 2100 Bioanalyzer (Agilent Technologies, Santa Clara, CA, USA), respectively. RNA was aliquoted and used for the four experimental approaches CoolMPS, BGISEQ, Illumina and reverse transcriptase-quantitative polymerase chain reaction (RT-qPCR) as described below. The study was approved by the ethical committee of the Medical Faculty of the University of Tuebingen (Nr. 90/2009BO2). A list of samples included in the study is available as Supplementary Table S1.

### CoolMPS™ on the DNBSEQ-G400RS

MiRNA libraries were prepared using the MGIEasy Small RNA Library Prep Kit (MGI Technologies, Shenzhen, China; product number 1000006383) with 800 ng total RNA input according to the manufacturer's recommendations. First, adapter sequences were ligated to the 3′ end of the RNA, followed by ligation of barcoded RT primers. Next, a universal adapter was ligated to the 5′ end. The RNA was then transcribed into cDNA by HiScript II Reverse Transcriptase in the presence of RNAse inhibitor. The primers used for the reverse transcription contained barcodes that allowed the pooling of up to 24 samples per sequencing library. Then cDNA libraries were amplified by 18-cycles of PCR reactions. Amplified PCR products were size selected using 6% TBE gel electrophoresis and the band from 100 to 120 bp was then purified with spin-X centrifuge tube filters followed by ethanol precipitation. The purified PCR products were quantified using Qubit dsDNA HS Assay kit (Invitrogen, Cat No. Q32854). Twelve purified PCR products were pooled with 84 fmol each (total 1 pmol) and circularized using a specific oligo sequence complementary to sequences in both the 3′ and 5′ adaptors provided in the MGIEasy Small RNA Library Prep Kit. The remaining linear DNA was digested. After purification, the single strand circularized DNA library was quantified using Qubit ssDNA Assay Kit (Invitrogen, Cat No, Q10212). Subsequently, DNA nanoballs (DNBs) were generated using rolling circle amplification from 60 fmol of single stranded, circularized DNA library for 25 min. The DNB concentration was determined using Qubit ssDNA Assay Kit. The DNBs (concentration in the range of 8–20 ng/μl) were mixed with loading buffer by manual pipetting and subsequently loaded onto DNBSEQ-G400RS 4-lane flowcells (product number 1000016985) using the MGIDL-200H DNB loader as described in the CoolMPSTM High-throughput Sequencing Set User Manual provided with the kit. Loaded flow cells were sequenced on the DNBSEQ-G400RS instrument using CoolMPS^TM^ SE50 beta sequencing kits, now available as commercial products (product number 1000019478, MGI Tech Co., Ltd, Shenzhen, China) following manufacturers recommendation. The MGI CoolMPS^TM^ SE50 kits are the standard product for small RNA sequencing. Sequencing was performed by selecting the smallRNA sequencing plan from the application menu on the DNBSEQ-G400RS. Single end sequencing of 50 bp along with 10 bp of barcode was performed. The basic difference between CoolMPS and standard sequencing-by-synthesis, relying on incorporation of labeled nucleotides, is the incorporation of unlabeled, reversibly terminated nucleotides. The fluorescent signal to detect the incorporated bases is generated by using base-specific 3′ block-dependent fluorescently labeled antibodies. After each cycle, the bound antibodies are removed and 3′ blocking moiety on the sugar group of the nucleotide regenerates the natural nucleotides. This procedure has the advantage not leaving a mark on the base and making the current sequencing cycle independent on the previous one. Base calling and generation of FASTQ files on the DNBSEQ-G400RS was performed using the software release for CoolMPS (BasecallLite version_1.0.7.84). An important machine quality control step included the removal of tiles from the FASTQ files that failed at some point in the base calling process leading to ‘N’ bases for all reads in that respective tile. A detailed description of the CoolMPS method and procedures is available under: https://doi.org/10.1101/2020.02.19.953307. The sequencing has been performed by Complete Genomics Incorporated, San Jose, California. The overall process of library preparation and sequencing on the DNBSEQ-G400 is referred to as ‘CoolMPS’ through the whole manuscript.

### BGISEQ-500 sequencing using standard cPAS

As described above for CoolMPS, the MGIEasy Small RNA Library Prep Kit (product number 1000006383) was used to generate circularized DNA libraries with 800 ng total RNA input according to the manufacturer's recommendations. The library preparation and DNB preparation procedures are exactly the same as the one described in the previous section. DNBs were loaded onto the flow cell using the BGIDL-50 DNB loader and single end 50 bp sequencing was performed using the BGISEQ-500RS High-throughput Sequencing Set SE50 on the BGISEQ-500RS instrument. The sequencing has been carried out in the Human Genetics Department at Saarland University, Germany. This process is referred to as ‘BGISEQ’ through the whole manuscript.

### Illumina library preparation and sequencing

Libraries were prepared according to the protocol of the TruSeq Small RNA Sample Prep Kit (Illumina) with 200 ng of total RNA per sample as starting material as described previously (24). In brief, the concentration of the libraries was assessed using a Bioanalyzer with the DNA 1000 Chip. Before sequencing, libraries were pooled in equal amounts of batches of six samples and clustered with a concentration of 9 pmol in one lane each of a single read flow cell. Sequencing of 50 cycles was performed on a HiSeq instrument (Illumina). Demultiplexing of raw sequencing data and generation of FASTQ files was performed with CASAVA v1.8.2.

### RT-qPCR

RT-qPCR experiments are described in detail in the original publication (25). In brief, the miScript PCR system was used with custom miRNA PCR arrays (all reagents from Qiagen, Hilden, Germany). The PCR arrays were designed in 96-well plates to measure the expression of human miRNAs and RNU48 as well as RNU6 as endogenous controls. The RT-qPCR experiments have been performed in the Human Genetics Lab of Saarland University. Reverse transcription was performed using 100 ng total RNA as input using miScriptRT-II kit in 20 μl total volume. PCR reactions with 1 ng cDNA input in a total volume of 20 μl were set up automatically using the miScript SYBR Green PCR system in a Qiagility pipetting robot (Qiagen, Hilden, Germany).

### Bioinformatics

The pre-processing of the FASTQ files of CoolMPS, BGISEQ and Illumina has been done using miRMaster 1.1 (16,26). MiRMaster is freely accessible at https://www.ccb.uni-saarland.de/mirmaster/. Briefly, adapters at the 3′ end were trimmed, while allowing an error of maximum one base and requiring a minimum overlap with the read of 10 bases. Reads were quality trimmed when the average quality dropped below 20 in a window of four consecutive bases to ensure a high quality of reads used for the downstream processing. All reads shorter than 17 bases after trimming were discarded from all further analyses. Read duplication levels were computed with FASTQC 0.11.8. The error rate per base was estimated by mapping the trimmed reads to the human genome with bowtie, while allowing up to three mismatches (command line: bowtie -v3 -k 1 –best –fullref) and counting the mismatched bases with Samtools stats (version 1.9, (27)). To further ensure the best comparability, BGISEQ and Illumina data were subsampled to match the CoolMPS distribution that was originally sequenced to a lower extent. In detail, all samples were subsampled to a read depth of 10 Million reads. Reads were mapped to the primary assembly of GRCh38.p10 using bowtie 1.2.2 (28), while allowing no mismatches and discarding reads mapping to over 100 locations (command line: bowtie -v0 -m 100 –best –strata –fullref). Read RNA classes were determined using FeatureCounts 1.5.2 (29) and annotations of GENCODE v25 (30), piRBase 1 (31), miRBase v22.1 and GtRNAdb 18.1 (32) with the following parameters: -F SAF –O –M –R –f –fracOverlap 0.9, which required an overlap of at least 90% of a read with the annotated region and allowed multimapping reads and overlapping features. MiRBase v22.1 miRNAs were quantified using miRMaster with up to one mismatch and a variability of two bases allowed at the 5′ end and five bases at the 3′ end. Novel miRNA candidates were predicted with miRMaster with a required minimum expression of five reads in at least 75% of all dementia or control samples. Since we expect numerous false positive hits from the next generation sequencing data we performed a quality control of the newly predicted candidates and evaluated them using the NovoMiRank tool (33). NovoMiRank was applied using the default parameters, i.e. miRBase versions 1–7 were used as reference to identify the most reliable candidates. All further downstream analyses have been carried out in R 3.6.1 (https://www.R-project.org/). To test whether miRNAs were normally distributed, Shapiro–Wilk tests were computed per miRNA using the shapiro.test function from the stats package. As hypothesis test, parametric *t*-test and non-parametric Wilcoxon Mann-Whitney (WMW) test were performed using the t.test and wilcox.test functions from the stats package. Statistical tests for group comparisons were carried out as two-tailed and un-paired tests. All *P*-values were subjected to adjustment for multiple testing by using the Benjamini–Hochberg approach through applying the p.adjust function from the stats package. To estimate the effect sizes, the area under the receiver characteristic curve (AUC value) and the Cohen's D effect size were computed using the R pROC package (1.15.0, (34)) and the R effsize package (0.7.4). Plots were generated with ggplot2 (3.1.0), cowplot (0.9.4), complexHeatmap (2.5.3, (35)), ggridges (0.5.1) and vioplot (0.3.5). To compute the most significant overlap between the CoolMPS and BGISEQ technology in terms of dementia biomarkers we employed the dynamic programming based DynaVenn approach (36). DynaVenn is freely accessible at https://www.ccb.uni-saarland.de/dynavenn. Functional categories were analyzed by miRNA set enrichment analysis with default parameters using miEAA 2.0 (37,38) with a list of the miRNAs sorted with respect to their effect sizes as input (with separate adjustment of categories and Benjamini–Hochberg adjustment procedure).

## RESULTS

### Study setup allowing to evaluate technical and biological aspects

**Figure 1.**
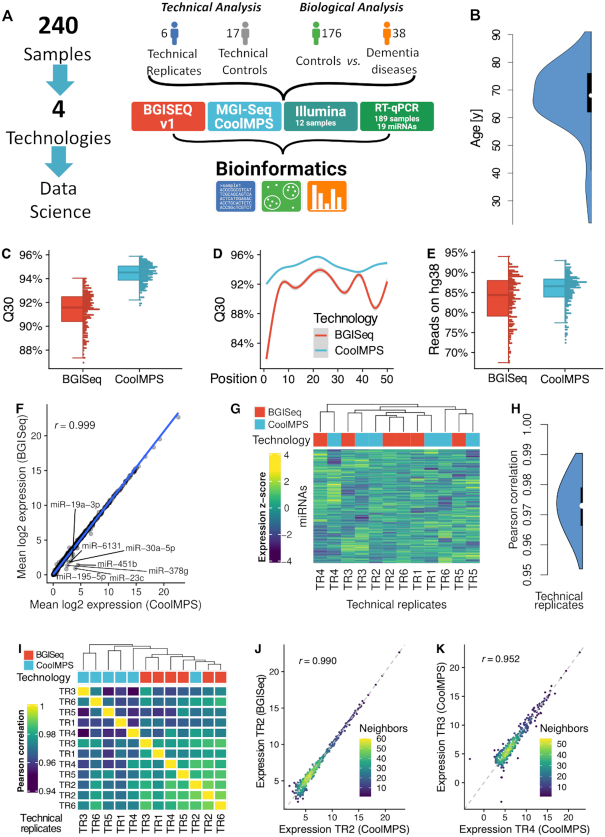
Study setup and quality control. (**A**) In the study we measured 240 individual blood samples using two fundamentally different sequencing approaches and compare the data by bioinformatics approaches before we compute the concordance to RT-qPCR profiles. The 240 samples include one part that has been used only for assessment of technical properties (6 and 17 samples in blue and gray) as well as a second part to evaluate performance related to biomarker discovery (176 controls in green and 38 dementia cases in orange). (**B**) Distribution of the age of the individuals included in the study, shown as violin plot. The black box spans the first to the third quartile and the white dot shows the median. (**C**) Distribution of the average Q30 value per sample for the two technologies, shown as boxplot (left) and dotplot (right). Each sample is shown as one dot. The boxes span the first to the third quartile with the horizontal line inside the box representing the median value. The whiskers show the minimum and maximum values or values up to 1.5 times the interquartile range below or above the first or third quartile if outliers are present. (**D**) Q30 value over all samples per technology as function of the position in the read. The smoothed curve is fitted by a generalized additive model using a cubic regression spline. The gray area represents the confidence interval of the fit. (**E**) Distribution of the percentage of reads mapping to the human reference genome hg38 without mismatch per technology, shown as boxplot (left) and dotplot (right). Each sample is shown as one dot. The boxes span the first to the third quartile with the horizontal line inside the box representing the median value. The whiskers show the minimum and maximum values or values up to 1.5 times the interquartile range below or above the first or third quartile if outliers are present. (**F**) Scatter plot of the average expression of all miRNAs in all samples for the two technologies. The blue line is the regression line. The Pearson correlation is shown in the upper left part of the plot. MiRNAs with a fold change larger than two between both technologies are highlighted. (**G**) Heat map of the clustered expression *z*-scores of miRNAs (rows) and technical replicates (columns). The color code for the columns represents the technology. The dendrogram shows the hierarchical clustering of the samples with Euclidean distance and complete linkage. (**H**) Distribution of all 12*11/2 = 66 pairwise Pearson correlation coefficients, shown as violin plot. The black box spans the first to the third quartile and the white dot shows the median. (**I**) Correlation matrix of the expression values of all miRNAs for all technical replicates. The dendrogram shows the hierarchical clustering of the samples with Euclidean distance and complete linkage. (**J**) Scatter plot of miRNAs for the best correlation between two technical replicates. The dotted line represents the angle bisector. The Pearson correlation is shown in the upper left part of the plot. The points are colored according to the point density in their neighborhood. (**K**) Scatter plot of miRNAs for the worst correlation between two technical replicates. The dotted line represents the angle bisector. The Pearson correlation is shown in the upper left part of the plot. The points are colored according to the point density in their neighborhood.

Primary aim of the study was to compare the combinatorial probe-anchor synthesis (cPAS)-based data using labeling of nucleotides to the data generated by the new antibody labeled-based approach on the more recent DNBSEQ-400RS systems. In the context of this manuscript, the former approach is referred to as BGISEQ and the latter as CoolMPS. Secondary aim was to compare the performance and comparability of both approaches in terms of potential liquid biopsy biomarker tests. We thus selected a study setup that allows to address both aims (Figure 1A). We sequenced 240 individual blood samples on both sequencing systems. The 240 samples include 179 controls and 38 patients with dementia. This part of the cohort has been used to evaluate the performance of both technologies to detect dementia biomarkers. Furthermore, the 240 samples include 17 individuals and 6 technical replicates. The latter samples were not used for the biomarker study but to assess the general stability and reproducibility of the technologies. Further, we compared the data to RT-qPCR measurements of a subset of 19 miRNAs in 189 samples and also evaluated the performance in comparison to data generated by Illumina sequencers. A full list of miRNAs and samples together with the respective Delta CT values from the RT-qPCR validation is available in Supplementary Table S2. We first evaluated the general performance of CoolMPS for quantification of RNA and then provide results of CoolMPS as liquid biopsy biomarker for dementia. The cohort was composed of participants with an average age of 67.3 years and a standard deviation of 12.3 years (Figure 1B). Details on the sequencing approaches and data analyses are given in the ‘Materials and Methods’ section.

### Key performance indicators reveal improved quality of CoolMPS

First, we compared the Q30 values for the reads from the two sequencing approaches (Figure 1C). The Q30 value provides the percentage of bases sequenced with a Phred score of at least 30, corresponding to an error rate of 0.1%. The median Q30 of the BGISEQ was 91.8% while the median Q30 of CoolMPS jumped to 94%, representing a significant improved performance of CoolMPS (*P* < 10^−10^). Intriguingly, we observed the higher per base sequencing accuracy over the complete read length not observing any drop at the beginning or at the end of the read. Moreover, CoolMPS showed lower variability in sequencing performance over the read in general as well as lower variability per base in the read (Figure 1D). While the variation of valid reads per sequencing run still varied for the CoolMPS technology we observed a constantly higher fraction of reads mapping without mismatches to the human genome (84.9% for BGISEQ and 86% for CoolMPS; Figure 1E). We also investigated the GC content of the generated libraries and found a median of 51.10% for BGISEQ and a median of 50.72% for CoolMPS in the unprocessed data, which dropped to a median of 42.38 and 41.60% for BGISEQ and CoolMPS after adapter and quality trimming, respectively (Supplementary Figure S1A and B). The mean quality scores per position varied between 33.95 and 36.35 for CoolMPS and even increased slightly toward the end of the read. In contrast, the BGISEQ reads varied between 27.95 and 36.17 and reached their peak at position 26. Then, the quality of BGISEQ reads decreased until position 50 (Supplementary Figure S1C and D). The mean quality scores for the trimmed files, i.e. those that did not contain any adapters, varied similarly, although the mean quality scores decreased more for longer reads. The estimated error rate was for both technologies similar with a median of 0.74% for BGISEQ and 0.76% for CoolMPS (Supplementary Figure S1E). For both, the raw sequencing files, and the trimmed ones, we observed a close to identical GC content distribution. For both technologies we observed two distinct peaks at 51 and 57% (Supplementary Figure S1F and G). We also found that the read length in both libraries after trimming peaked at 22, as we expected from a miRNA enriched library (Supplementary Figure S1H). We further evaluated the duplication levels of the CoolMPS and BGISEQ libraries. In both cases, the distributions were again nearly identical, showing most duplication levels above 10 000 (Supplementary Figure S1I and J). This is expected from miRNA libraries, as often a small number of miRNAs account for most of the reads. Finally, we checked the read base composition and found similar patterns. The first 22 bases reveal the most overrepresented sequence (i.e. the sequence of hsa-miR-451a), followed by the bases of the adapter sequence for the raw reads, and by less sequence specific bases for the trimmed reads (Supplementary Figure S1K and L). For most of the tested relevant key performance indicators (e.g. Q30 and reads mapping to the human genome) that allow to compare the general sequencing performance, CoolMPS yielded an increased performance compared to the classical BGISEQ approach.

Next, we evaluated and compared the reproducibility of the two technologies. When comparing the mean expression of all samples for CoolMPS to BGISEQ we obtained an extremely high correlation of 0.999 (Figure 1F). The scatter plot highlights a set of seven miRNAs, which were measured with higher expression in the CoolMPS data as compared to BGISEQ (miR-19a-3p, miR-30a-5p, miR-6131, miR-451b, miR-378g, miR-195-5p and miR-23c). Next, we considered only the six technical replicates per technology. There, these miRNAs reveal the same pattern as for the complete set of samples, thus excluding variance related to the disease status of the participants as potential cause (Supplementary Figure S2). Sequence and structure properties of these miRNAs are shown in Supplementary Table S3. Neither the length, nor the base composition or secondary structures reveal a joint pattern, arguing against a technological bias. We then asked whether we observe a clustering according to the sequencing approach or whether CoolMPS and BGISEQ samples mix. Indeed, hierarchical clustering indicates that the samples do not cluster by technology (Figure *1**G*). The Pearson correlation between all *12 × 11 / 2 = 66* pair wise comparisons of technical replicates varied between 0.952 and 0.990 with a median performance of 0.973 (Figure 1H). The correlation matrix revealed marginal differences in the correlation coefficients between all the BGISEQ replicates (median 0.980) in comparison to the ones between the CoolMPS samples (median 0.964) (Figure 1I). Also, the correlation between the two technologies with a coefficient of 0.973 was high. The differences in the correlation lead to a tendency of technologies to cluster together, although CoolMPS Technical Replicate 2 clustered with BGISEQ Technical Replicates 2 and 6. Scatter plots for the best (Figure 1J) and the worst correlation (Figure 1K) demonstrate the generally very high reproducibility between the technologies that is in the same range as technical replicates within the technologies. Most importantly, we did not observe any significant change between the RNAs profiled with BGISEQ compared to CoolMPS after adjustment for multiple testing, both, for the WMW and the *t*-test.

Having understood basic performance of the sequencing technology as well as core aspects on technological reproducibility we next evaluated the content of the different sequencing approaches with respect to quantitative and qualitative aspects.

### Composition of different RNA classes is similar between BGISEQ and CoolMPS

**Figure 2.**
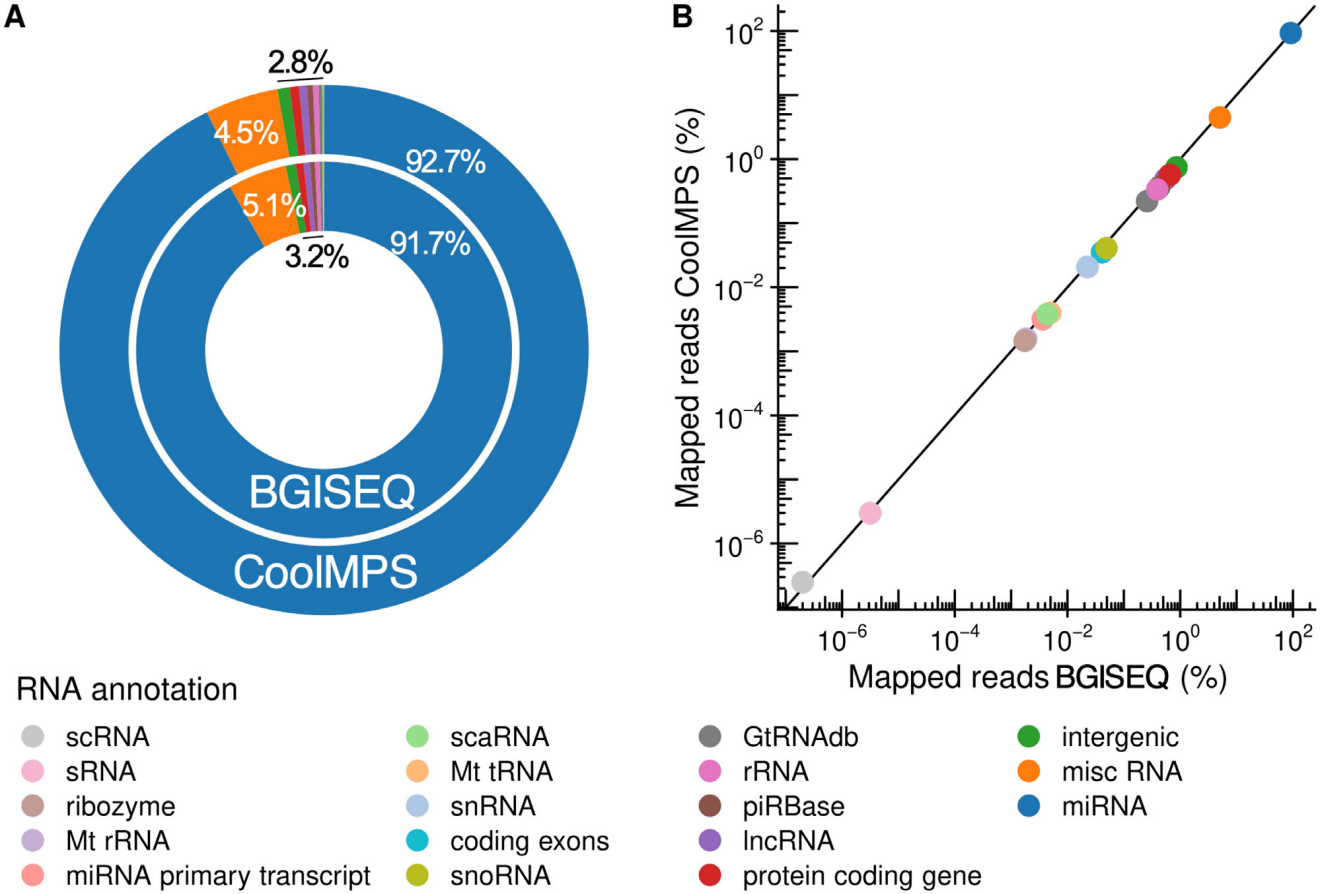
Distribution to the different sncRNAs classes. (**A**) Donut plot comparing the distribution of all RNA classes and intergenic regions that were covered by reads from CoolMPS and BGISEQ. (**B**) Scatter plot that shows the percentage of reads mapping to the RNA classes and intergenic regions for BGISEQ (*x*-axis) and CoolMPS (*y*-axis) on a logarithmic scale.

The first question related to small non-coding RNA sequencing data is the representation of different RNA classes. Different sample- and library preparation protocols lead to varying results. For example, size selection is applied to enrich-specific populations of sncRNAs. To minimize respective effects and to focus on the performance of the sequencing technique, we used the same libraries for sequencing and purified small non-coding RNAs by gel electrophoresis (see ‘Materials and Methods’ section). This protocol has been optimized to enrich for miRNAs, however, leaving also reads to evaluate other RNA classes. The distribution to the different classes matched generally very well between BGISEQ and CoolMPS (Figure 2A). Especially, we observed the intended enrichment for miRNAs. For BGISEQ, 91.7% of all mappable reads matched to miRNAs, for CoolMPS we even reached a higher mapping of 92.7%. The second most abundant RNA class was the Ensembl's misc RNA category, containing among others yRNAs and signal recognition particle RNAs (SRP RNAs). This category contains 5.1% of all BGISEQ and 4.5% of all CoolMPS reads. All other categories were covered by less than 1% of reads in both technologies. The scatter plot contrasting the log_10_ percentages for both technologies highlights the very reproducible distribution of reads to the different RNA classes (Figure 2B). Since the protocol was optimized to enrich for miRNAs and our results demonstrate that this enrichment was successful, we focused on comparing the performance for this class of sncRNAs.

### CoolMPS yields more novel miRNA candidates

**Figure 3.**
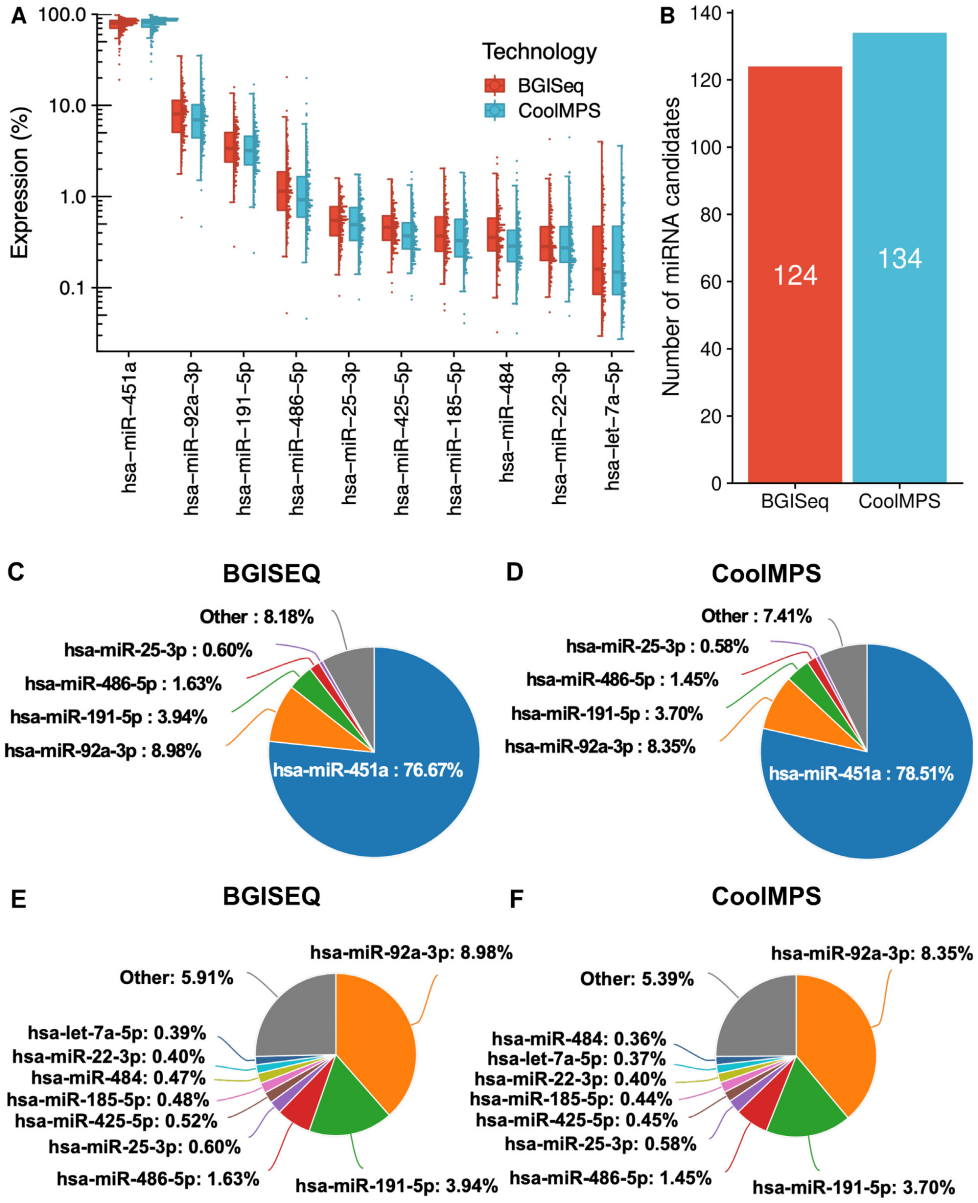
Distribution to microRNAs. (**A**) Distribution of the read percentage of the 10 most abundant miRNAs in the CoolMPS and BGISEQ data, shown as boxplot (left) and dotplot (right). Each sample is shown as one dot. The boxes span the first to the third quartile with the horizontal line inside the box representing the median value. The whiskers show the minimum and maximum values or values up to 1.5 times the interquartile range below or above the first or third quartile if outliers are present. (**B**) Number of novel microRNA candidates for both technologies. (**C**) Pie chart for the top five miRNAs on the BGISEQ. (**D**) Pie chart for the top five miRNAs on the CoolMPS. (**E**) Pie chart for the top ten miRNAs on the BGISEQ after exclusion of the most abundant miR-451a. (**F**) Pie chart for the top ten miRNAs using CoolMPS after exclusion of the most abundant miR-451a.

With respect to different technologies a bias in sncRNA-seq data is known. Especially for specimen types such as whole blood where already an enrichment of selected miRNAs exist, additional technological bias can further impair the data analysis. In whole blood, miRNA expression is not uniformly distributed but few miRNAs are significantly higher expressed than others. Technology bias further overamplifies the respective miRNA reads. These circumstances complicate the discovery of new miRNAs with the aim of completing the repertoire of annotated miRNAs (8). We thus evaluated and compared the distribution of reads to different miRNAs using the two sequencing technologies and asked how many novel miRNA candidates could be obtained. As expected, we observed an uneven distribution, which is however highly concordant between the technologies (Figure 3A). At the same time, we discovered 124 novel miRNA candidates using BGISEQ while CoolMPS based results highlight 134 novel miRNA candidates (Figure 3B and Supplementary Figure S3A). These findings suggest a higher sensitivity in terms of discovering low abundant yet unknown miRNA molecules. Remarkably, a large fraction of all new microRNA candidates, in total 88, have been detected by both technologies. To assess the quality of those miRNA candidates we scored them using NovoMiRank. The score computed by NovoMiRank considers sequence and structural features and describes the average distance of the new candidates to a reference set, which is per default miRBase v1-7. The median score obtained for the common candidates was 1.12, while the technology specific candidates obtained median scores of 1.05 for CoolMPS and 1.00 for BGISEQ. As highlighted by the distribution shown in Supplementary Figure S3B, the score ranges are similar between the approaches and only few candidates (four detected by both CoolMPS and BGISEQ, three CoolMPS specific and one for BGISEQ specific) showed scores above 1.5. The score of 1.5 has been set since it is the maximum score observed for miRBase v1-7 miRNAs i.e. the reference set of NovoMiRank. In summary, both technologies do not reveal quantitative differences in the quality of reported miRNAs but only in the quantity, with remarkable advantages of CoolMPS.

In comparing the distribution of miRNAs annotated in the miRBase we observe 76.7% of all BGISEQ reads mapping to the most abundant miRNA (miR-451a; Figure 3C). Using CoolMPS, 78.5% of all reads matched to this miRNA (Figure 3D). The second most abundant miRNA is represented by 9 and 8.4% of all reads, respectively (miR-92a-3p). In sum, the top five miRNAs are covered by 93.8% of all reads in the BGISEQ and by 92.6% of all reads in the CoolMPS approach. A more detailed breakdown by excluding the most abundant miR-451a demonstrates that the order of the 10 most abundant miRNAs matches perfectly between the two technologies (Figure 3E and F). At the same time, the data reinforces that especially for biospecimens with an uneven distribution of miRNA molecules, deep sequencing with the least possible bias is required to profile known and to discover new miRNAs.

### Comparing biomarker profiles shows high reproducibility between the different approaches

**Figure 4.**
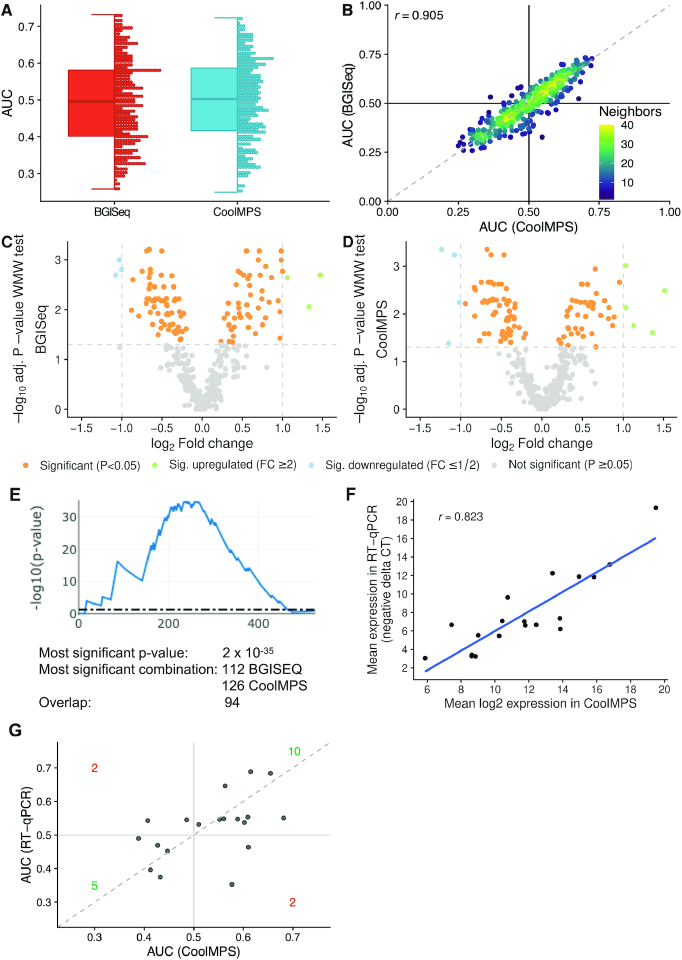
Diagnostic performance on dementia patients. (**A**) Distribution of the AUC values to differentiate between dementia and controls obtained for both technologies. An AUC of 0.5 means no dys-regulation. A deviation from 0.5 toward one means an upregulation and toward zero a downregulation of the biomarkers. The distribution is shown as boxplot (left) and dotplot (right). Each miRNA is shown as one dot. The boxes span the first to the third quartile with the horizontal line inside the box representing the median value. The whiskers show the minimum and maximum values or values up to 1.5 times the interquartile range below or above the first or third quartile if outliers are present. (**B**) Scatter plot of the AUC values to differentiate between dementia and controls in CoolMPS (*x*-axis) versus BGISEQ (*y*-axis). The black horizontal and vertical line represent the AUC value of 0.5, respectively. The Pearson correlation is shown in the upper left part of the plot. The points are colored according to the point density in their neighborhood. (**C**) Volcano plot showing the log_2_ fold change on the x-axis and the FDR adjusted negative log_10_ of the Wilcoxon–Mann–Whitney (WMW) *P*-value on the *y*-axis for BGISEQ. Orange dots are located above the horizontal line and are significant. Blue and green dots above the horizontal and on the left / right of the vertical lines are significant and have a fold-change above 2. (**D**) Same volcano plot as in Figure 4C, but for CoolMPS. (**E**) Result of DynaVenn that presents the negative log_10_ of the overlap between the two miRNA sets dependent on how many miRNAs are included. The peak of the curve represents the most significant overlap. (**F**) Scatter plot of the log_2_ CoolMPS expression (*x*-axis) and the negative delta CT value for the 19 miRNAs included in the validation study. The Pearson correlation coefficient is shown in the upper left part of the plot. (**G**) Scatter plot of the AUC values to differentiate between dementia and controls in CoolMPS (*x*-axis) and BGISEQ (*y*-axis). The dashed line represents the angle bisector.

One of the most important question in introducing new technologies is not only whether general performance improves but also whether previous biological results can be reproduced. One core example are biomarker tests. Often, biomarker sets change substantially when a new quantification approach is introduced. This might be an expected and even desired result, e.g. if a new technology generation with higher technical sensitivity is introduced. But if a new technology has the main task to support translation of biomarkers to care by facilitating better integration into clinical workflows or lower experimental costs, original biomarker profiles should not be compromised. We thus evaluated the diagnostic performance of miRNA biomarkers using BGISEQ and CoolMPS and used a liquid biopsy dementia test as validation example. We sequenced cases with dementia as well as controls with similar age distribution (Figure 1A and B). As performance criteria we considered the result of two commonly used hypothesis tests, the *t*-test and the WMW test. Since not all miRNAs were normally distributed according to the Shapiro Wilk test, we here focus on the results of the WMW test and provide the t-test *P*-values only in the supplement (Supplementary Table S4 and 5). Because of known challenges with *P*-values and the controversial discussion on this topic (39), we also computed effect sizes, namely Cohen's D and the area under the receiver characteristics curve AUC. Detailed results for each miRNA and each of the different metrics are provided for both BGISEQ (Supplementary Table S4) and CoolMPS (Supplementary Table S5). In terms of AUC, BGISEQ and CoolMPS showed an almost identical distribution (Figure 4A). The scatter plot displays a very high degree of reproducibility (Pearson correlation coefficient of 0.905) between the two technologies considering the diagnostic performance (Figure 4B). As consequence, also the volcano plots for the two technologies were very similar (Figure 4C and D). Given the general concordance of the results we speculated that also the ranks of biomarkers were consistent between the two technologies. For the top-10 markers of BGISEQ and CoolMPS we thus compared the ranks and absolute values (Table 1). First, we recognized that the top marker performed better in CoolMPS as compared to BGISEQ in all metrics, the raw *P*-value, the adjusted *P*-value, the Cohen's D and the AUC. The adjusted *P*-values were for example 0.0006 in BGISEQ data and 0.0004 in CoolMPS data. Second, we observed that four miRNAs were among the top 10 markers in both technologies (miR-3200-3p, let-7e-5p, miR-15b-5p, miR-19b-3p). For other markers we computed partially very different ranks. One of the most extreme examples is miR-3335-5p, which is ranked 9th most significant in CoolMPS and 117th in BGISEQ. Nonetheless, this miRNA was significant in both approaches. On the one hand we observed a very high correlation, on the other hand, we also noticed substantial differences in the ranks, most likely related to the close range of the *P*-values, challenging the concept of fixed thresholds. To overcome the bias of selecting fixed rank ranges, we developed the DynaVenn approach that computes the most significant overlap between two biomarker sets containing technical or biological replicates. DynaVenn computed the best overlap in selecting the best 112 miRNAs from BGISEQ and the best 126 miRNAs from CoolMPS, yielding an overlap of 94 miRNAs and a *P*-value of 2 × 10^−35^ (Figure 4E). Thus, the two biomarker sets show a highly significant overlap which might have remained hidden if only the top 10 markers would have been considered.

**Table 1.** For the top 10 most significant miRNAs with both technologies the rank in each technology is provided, followed by nominal and adjusted *P*-value, the effect size (Cohen's D) and AUC

miRNA	Rank BGISEQ	Rank CoolMPS	WMW raw *P*-value	WMW adj *P*-value	Cohen's D	AUC
**hsa-miR-3688-3p**	1	16	2.89E-06	0.0006	−0.88	0.26
**hsa-miR-3200-3p**	**2**	**4**	**3.23E-06**	**0.0006**	**−0.87**	**0.26**
**hsa-let-7d-5p**	3	17	6.98E-06	0.0007	0.83	0.73
**hsa-miR-589-5p**	4	51	9.26E-06	0.0007	−0.79	0.27
**hsa-miR-550a-3-5p**	5	NA	9.39E-06	0.0007	0.78	0.73
**hsa-let-7e-5p**	**6**	**6**	**1.06E-05**	**0.0007**	**0.82**	**0.73**
**hsa-miR-193a-3p**	7	69	1.21E-05	0.0007	−0.76	0.27
**hsa-miR-4448**	8	55	2.21E-05	0.0010	0.57	0.72
**hsa-miR-15b-5p**	**9**	**8**	**2.55E-05**	**0.0010**	**0.77**	**0.72**
**hsa-miR-19b-3p**	**10**	**1**	**2.64E-05**	**0.0010**	**−0.77**	**0.28**
**hsa-miR-181c-5p**	21	2	2.38E-06	0.0004	−0.80	0.26
**hsa-miR-185-5p**	48	3	4.84E-06	0.0006	−0.75	0.26
**hsa-miR-5695**	12	5	7.70E-06	0.0006	−0.76	0.27
**hsa-miR-363-3p**	33	7	2.15E-05	0.0011	0.75	0.72
**hsa-miR-335-5p**	117	9	5.55E-05	0.0022	−0.44	0.29
**hsa-miR-30b-5p**	83	10	5.83E-05	0.0022	−0.72	0.29

Bold miRNAs are in the top 10 for both technologies.

### Illumina sequencing data shows differently biased but comparable measurements

In addition to BGISEQ we also compared the performance of CoolMPS to standard Illumina sequencing for small non-coding RNAs on a subset of 12 samples (24). As part of our quality control we filter reads shorter than 17 nucleotides. We thus compared the fraction of filtered reads for the three technologies on the subset of samples sequenced by the three technologies. For BGISEQ, 2.76% (SD of 0.56) of reads, for CoolMPS 3.90% (SD of 0.54) of reads and for Illumina 3.95% (SD of 3.24%) of reads were excluded. In a first analysis step we evaluated the Q30 values obtained by both approaches and found a median Q30 of 94.95% for Illumina in comparison to 93.10% for CoolMPS (Supplementary Figure S4A). The quality profile revealed Q30 values going up to a median of 99.43% for Illumina in the first 20 positions, whereas a strong drop could be observed afterwards, going down to a Q30 of 87.33% at position 50 (Supplementary Figure S4B). In comparison, the CoolMPS quality remained more stable for the complete read length with an average Q30 of 93.12% (SD: 1.79%) and even showed an increased quality toward the end of the reads. For the fraction of reads that can be used in further analyses, i.e. the ones mapping to the human genome, we observed for CoolMPS a median of 90.74%, while for Illumina only 77.85% could be mapped (Supplementary Figure S4C). In the next step, we inspected the expression similarity of both technologies and found a general agreement of both with a Pearson correlation of 0.873 (Supplementary Figure S4D). Nevertheless, we could observe 52 miRNAs with expression values differing by fold changes above 10, showing the technological specific biases (e.g. hsa-miR-486-5p was expressed 76 times higher in the Illumina samples). In addition, we confirmed that the samples of both technologies clustered separately according to their miRNA profiles (Supplementary Figure S4E) and showed a much higher intra-technology expression correlation (Pearson correlation of 0.960 for CoolMPS on median, 0.955 for Illumina) than between technologies (median Pearson correlation of 0.742) (Supplementary Figure S4F and G). We then asked if the RNA class distribution between both technologies show similar patterns. We found that the CoolMPS samples showed a higher diversity of RNA classes, whereas the Illumina samples contained a higher percentage of reads mapping to piRNAs (0.70 versus 0.17% in CoolMPS) and miRNAs (97.76 versus 94.98% in CoolMPS) (Supplementary Figure S5). Next, we focused on the composition of the detected miRNAs and found that of the top 10 most expressed miRNAs of both technologies, six overlapped. The largest differences could be observed for hsa-miR-486-5p and hsa-miR-451a, which are both the most expressed miRNAs in Illumina and CoolMPS and differ by a fold change of 76 and 87, respectively (Supplementary Figure S6A). For the Illumina samples, thus only 9.48% of the reads could be mapped to other miRNAs and after excluding the top 5 miRNAs, only 3.15% of the reads mapped to others (Supplementary Figure S6B). For the CoolMPS samples, we observed slightly increased mapping rates to the top five miRNAs on this subset of samples, with 5.60% of the reads mapping to the other miRNAs (Supplementary Figure S6C). Supplementary Figure S6D and E show a detailed breakdown of the top expressed miRNAs, after excluding the most abundant one. We also found that some miRNAs that were detected with low abundance in one technology (e.g. hsa-miR-223-3p and hsa-miR-185-5p for Illumina and hsa-miR-142-5p for CoolMPS) were among the 10 most expressed miRNAs in the other. This reinforces the necessity of deep sequencing, especially for the Illumina libraries, to quantify a larger range of miRNAs.

### RT-qPCR data largely fit to the CoolMPS measurements

Finally, it is important to understand whether a third and independent technology validates the biomarker profiles. Since we previously already validated the BGISEQ approach using RT-qPCR (23) and demonstrate in the present work that CoolMPS is concordant to BGISEQ we can speculate that the RT-qPCR data would also match the CoolMPS profiles. To evaluate this hypothesis, we compared the expression values of 19 miRNAs that have been measured for 189 samples from the present study by RT-qPCR (25). Between the mean log_2_ CoolMPS expression and the negative delta CT values computed from RT-qPCR we observed a high correlation of 0.823 (Figure 4F). To validate how well this translates into biomarker patterns we again computed the difference between controls and dementia patients (Figure 4G). In this comparison we observed 10 miRNAs that were upregulated in both technologies, 5 miRNAs that were downregulated in both technologies and four miRNAs that were discordantly regulated between the technologies. According to Fishers Exact test this corresponds to a significant overlap (*P* = 0.022).

### BGISEQ and CoolMPS AD miRNAs are matching known AD miRNAs and correlated to functional categories

As described in the previous sections, the miRNAs identified by the CoolMPS and BGISEQ approach have a significant diagnostic potential from a statistical perspective. We asked whether the signatures matched previously published results and which functional categories are enriched. To this end, we employed a miRNA set enrichment analysis using miEAA (37,38). As input the miRNAs were sorted with respect to their CoolMPS effect sizes. Downregulated miRNAs were most significantly associated to the miEAA disease category ‘Downregulated in Alzheimer's Disease’ (raw and adjusted *P*-value of 2.3 × 10^−5^ and 6.88 × 10^−4^) while upregulated miRNAs were most strongly correlated to glioma (raw and adjusted *P*-value of 0.002 and 0.025, respectively). With respect to Gene Ontology and pathway databases we computed two significant categories. Upregulated AD miRNAs were enriched in chromosome condensation (raw and adjusted *P*-value of 3.3 × 10^−6^ and 0.018) as well as response to magnesium ion (raw and adjusted *P*-value of 1.3 × 10^−5^ and 0.036).

## DISCUSSION

Whenever new technologies emerge in a field it is mandatory to test the fit to former technologies. The more disruptive a technological change is, the more the results differ from previous ones. An extreme example is the step from microarrays to RNA sequencing for analyzing expression profiles. If a novel technology aims to improve a previous one in a rather evolutionary manner by adapting and improving a specific step, the research results should generally be more aligned with previous findings. In biomedicine, such improvements can aim at an improved translational aspect of research in making workflows easier to use or in reducing the cost of assays. With CoolMPS we evaluated such an evolutionary improvement. Still, the main principle is sequencing-by-synthesis and also the detection and evaluation approach stay the same. The main difference is in using labeled antibodies instead of incorporating labeled nucleotides. While theoretical advantages of this approach, e.g. a potential re-use of the sequencing chemistry, are obvious we don’t expect disruptive new findings. It is essential to benchmark CoolMPS to related high-throughput approaches, in our case standard cPAS sequencing-by-synthesis and Illumina sequencing, but also to a gold standard technology, in our case RT-qPCR. As primary comparison high-throughput technology we selected cPAS on the BGISEQ since we already previously performed a detailed benchmarking to the Illumina sequencing-by-synthesis approach, highlighting the advantages and disadvantages of both approaches (23). As biospecimens we intentionally selected whole blood. Not only because whole blood samples can be used to screen for minimally invasive biomarkers but also because of their challenging characteristics. The repertoire of small non-coding RNAs varies between different blood cell types and sncRNAs have a very high dynamic range. In fact, this means that few high abundant molecules are sequenced often whereas low abundant molecules are hardly observed. In whole blood small non-coding RNA sequencing data generated by Illumina sequencers, partially over 90% of the reads belong to miR-486-5p. While this miRNA is certainly highly abundant in red blood cells, this extreme distribution does not seem to match reality. In both, the BGISEQ and CoolMPS data we still observe an extreme distribution with around ¾ of all reads matching to the most abundant miRNA, miR-451a. This can also be recognized in Supplementary Figure S1K and L. Still, this distribution is less extreme than for the previously investigated Illumina sequencing data. The less extreme overrepresentation in the BGISEQ and CoolMPS data thus facilitates the discovery of yet unknown and less abundant non-coding RNA molecules.

Among the top 10 markers that we discovered by CoolMPS (Table 1), eight miRNAs (miR-19b-3p, miR-181c-5p, miR-185-5p, miR-3200-3p, let-7e-5p, miR-15b-5p, miR-335-5p and miR-30b-5p) were already described in the literature to be correlated to Alzheimer's disease or dementia. For example, miR-19b-3p prevents amyloid β-induced injury by targeting BACE1 in SH-SY5Y cells (40) and is altered in CSF exosomes of AD patients (41). Similarly, miR-185-5p is known as exosomal AD biomarker (42). Also, let-7e-5p and miR-3200-3p were previously identified as blood biomarkers (43). Interestingly, the same manuscript also lists miR-30c-5p, miR-30d-5p and miR-15a-5p. For these miRNAs we report differential expression in related miRNA family members (miR-30b-5p and miR15b-5p respectively). The latter miRNA has also been reported in other studies a circulating AD biomarker (44,45) and targets the amyloid precursor protein (46). Similarly, miR-335-5p inhibits β-Amyloid in AD (47). Already for the 10 most significant miRNAs we thus found substantial evidence for their role in AD, both as biomarker but also linked to a potential pathogenic function.

One step in our analysis pipeline is to filter out short reads (below 17 nucleotides), that might add noise to the data. For BGISEQ, the lowest number of reads was filtered out in this step followed by CoolMPS and lllumina sequencing data. While the percentages overall were similar, we observed a higher standard deviation in Illumina data (3.24%) as compared to BGISEQ (0.54) and CoolMPS (0.56) data. In comparing CoolMPS data to Illumina data we observed a slightly better averaged Q30 value for the Illumina data. This advantage could be observed however mostly in the beginning of the read. Toward the end of the 50 base reads, Illumina Q30 values dropped more as compared to the stable performance of CoolMPS. This resulted in a higher mapping rate of the CoolMPS data. One explanation for a drop of quality is in the small size of miRNAs that are usually shorter than 25 nucleotides but 50 bases are sequenced. This effect might be more pronounced for Illumina as compared to the BGISEQ and CoolMPS data. In consequence, we can expect that this factor is likely less relevant for longer RNAs or sequencing of DNA. Also, the composition of the RNA classes was different between the technologies. Illumina data revealed higher percentages of piRNAs and miRNAs while CoolMPS shows a higher diversity also including other non-coding RNA classes. A difference between the BGISEQ/CoolMPS and Illumina protocols was the amount of starting material. For BGISEQ and CoolMPS, 800 ng was used while the Illumina data have been generated from 200 ng input material. This might pretend that a higher input amount is required for CoolMPS as compared to Illumina. We used this higher input amount however only during the exploratory phase of the CoolMPS protocol. Even with lower amount of input material down to 100ng we did not observe significant changes (data not shown). Indeed, the manufacturer's instruction would even allow input from 10ng RNA only. Thus, the input volume seems not to be a limiting factor for the CoolMPS technology.

In sum, both of the technologies have their advantages and disadvantages and the best systems should be chosen dependent on the application. Our data thus clearly suggest that small RNA sequencing results from Illumina data should not be directly compared to sequencing results from BGISEQ since the technical differences between identical samples are statistically highly significant. With respect to comparing between BGISEQ and CoolMPS datasets we observed generally very similar performance. The most striking advantage of CoolMPS is a significantly improved single base call quality. This led to marginal improvements in the biomarker patterns but did not improve the performance of any biomarker in a substantial manner. Interpreting the results, we have to bear in mind that the BGISEQ technology and chemistry have already matured over at least five years while we used prototype beta testing chemistry for CoolMPS. Since already this chemistry lead to improved performance we can expect further improvements with revised kits of CoolMPS. Finally, one big advantage is the potential to recover the used labeled antibodies for a second sequencing run.

## DATA AVAILABILITY

All sequencing data have been deposited in the Sequence Read Archive with the accession SRP271972.

## Supplementary Material

gkaa1122_Supplemental_Files
