# Supplementary material for: CoolMPS: evaluation of antibody labeling based massively parallel non-coding RNA sequencing
Source: Nucleic Acids Res. 2020 Dec 8;49(2):e10. doi: 10.1093/nar/gkaa1122 (PMC7826284; doi:10.1093/nar/gkaa1122)
Supplement: gkaa1122_Supplemental_Files [file gkaa1122_supplemental_files.zip › Supplemental Figures-.pptx]

## Slide 1
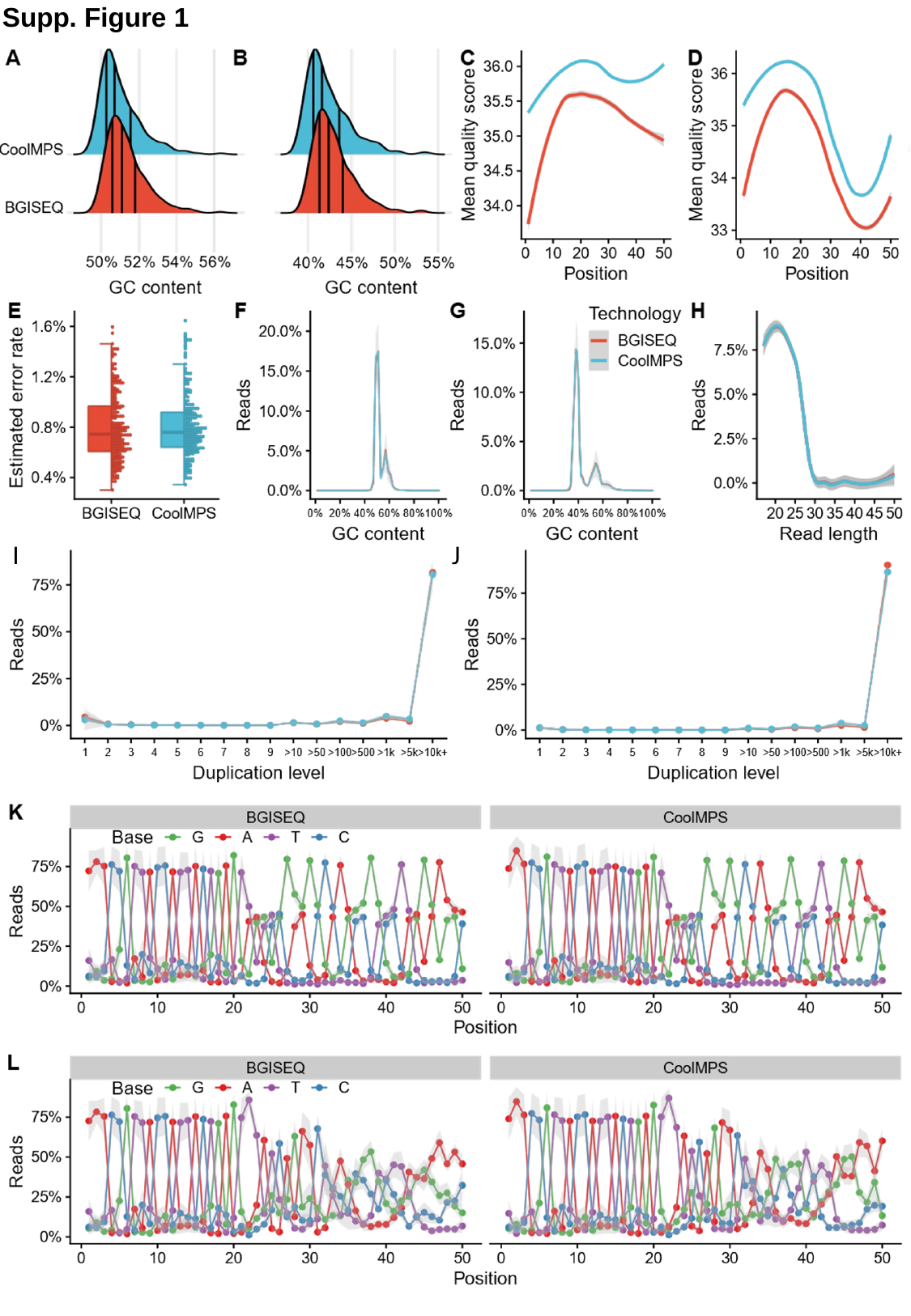

Supp. Figure 1
I
J

## Slide 2
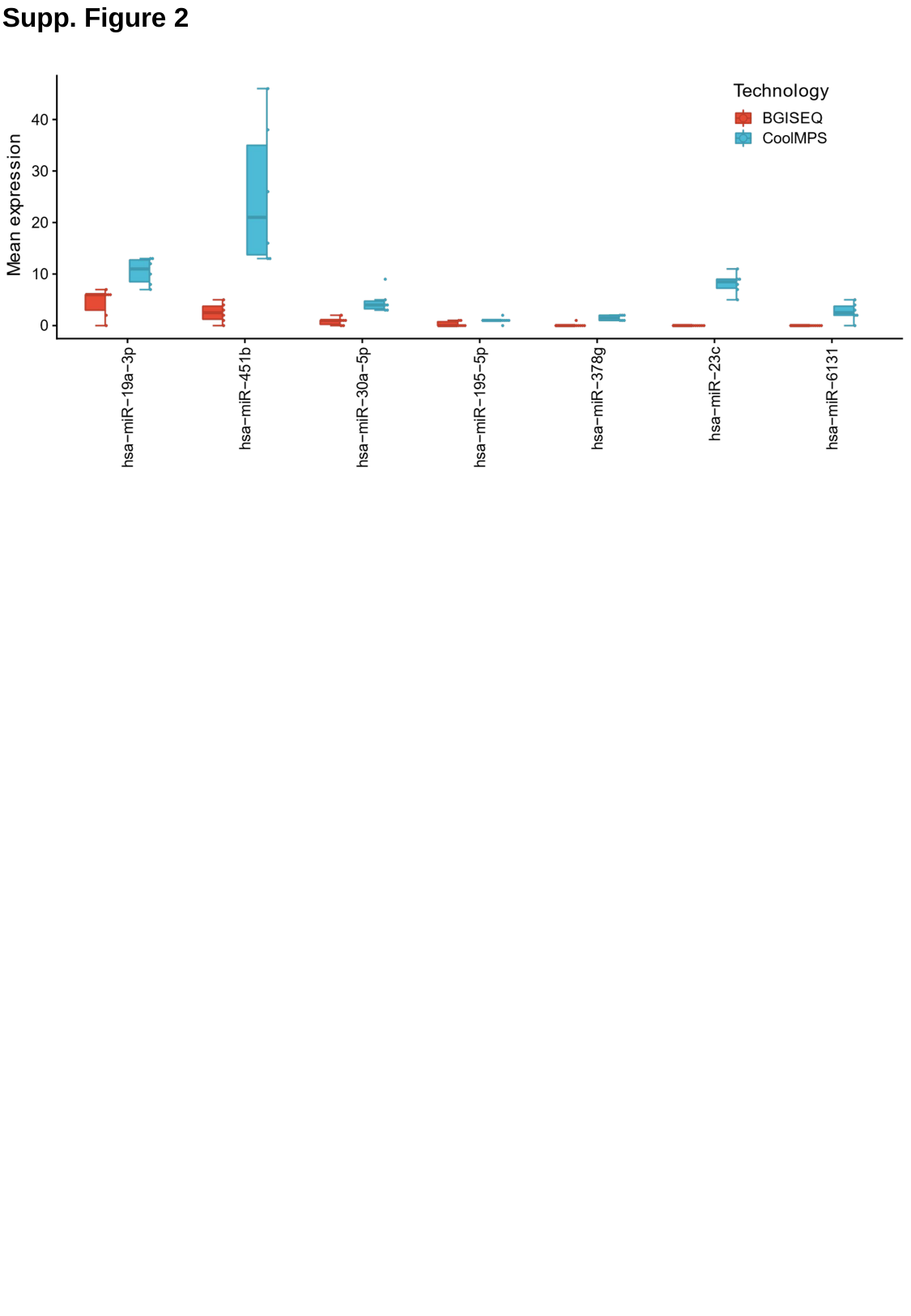

Supp. Figure 2

## Slide 3
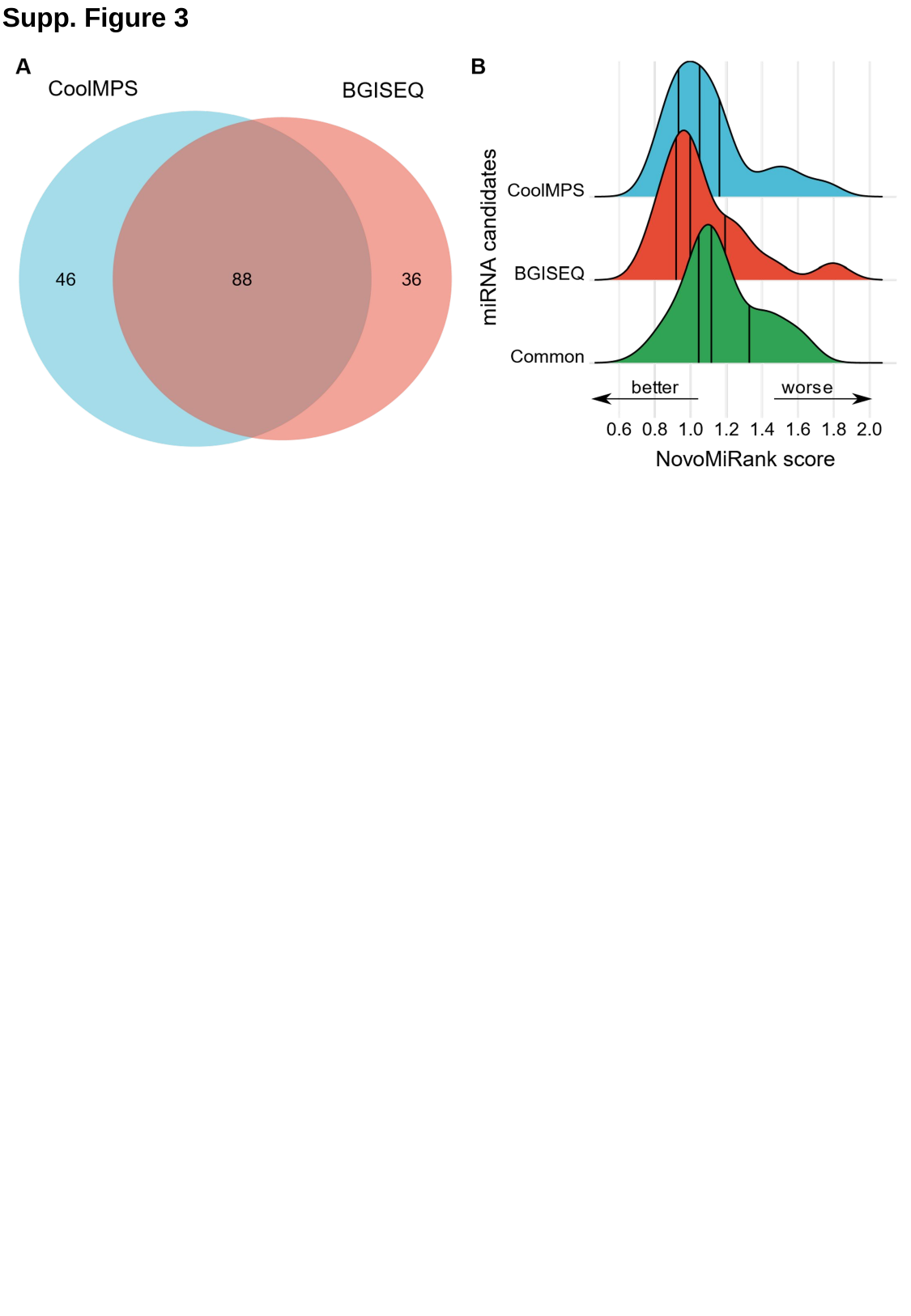

Supp. Figure 3

## Slide 4
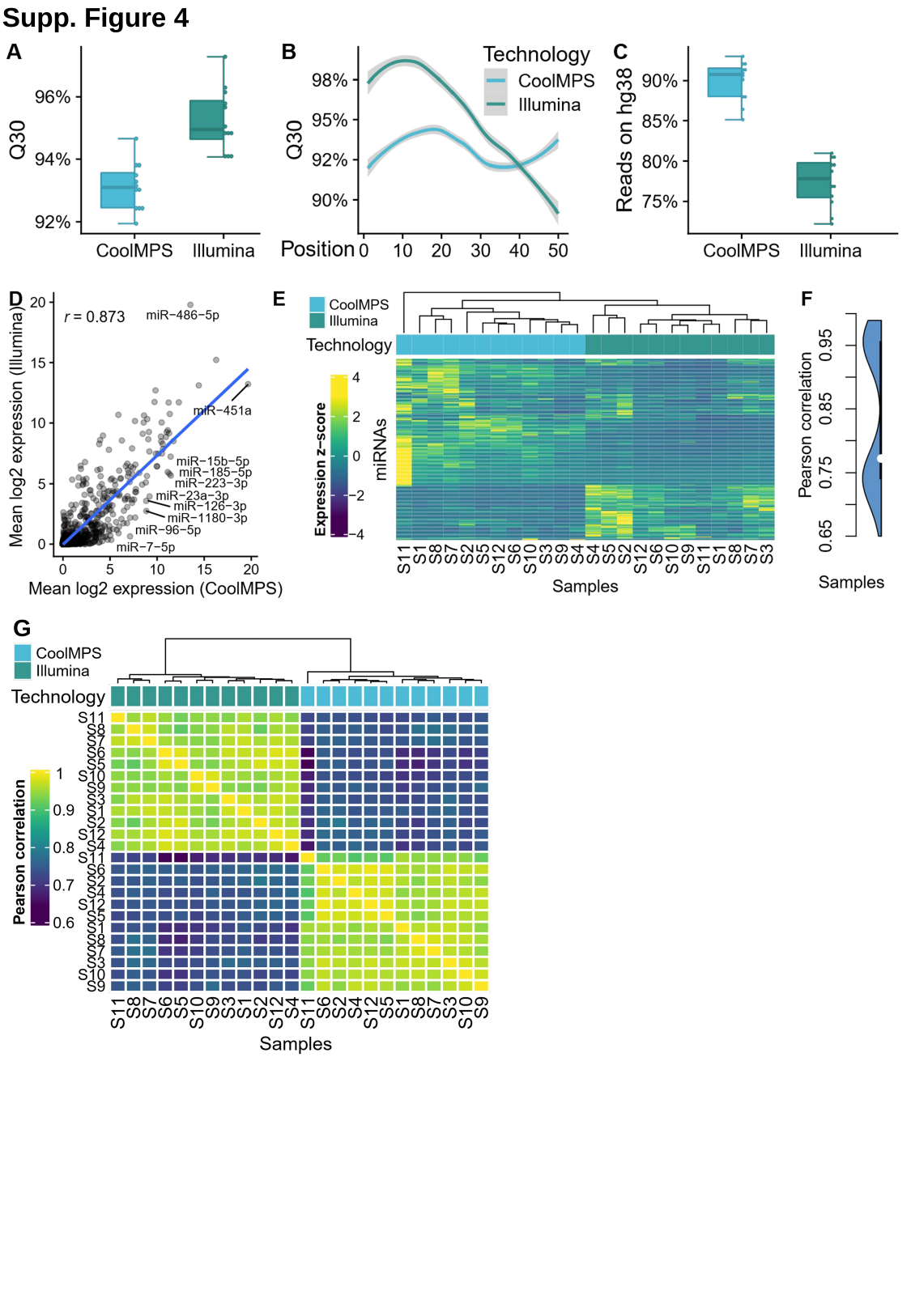

Supp. Figure 4
G

## Slide 5
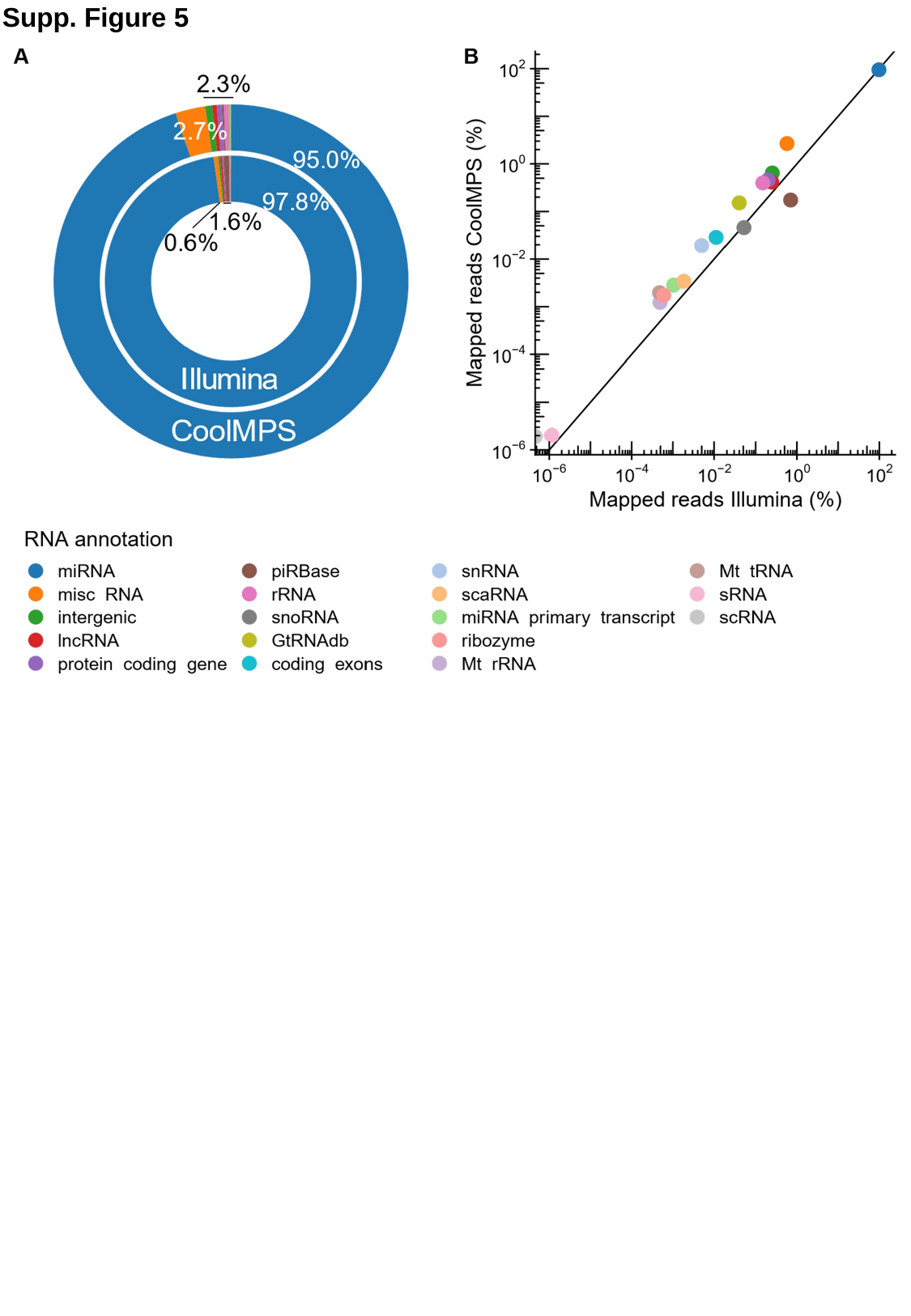

Supp. Figure 5

## Slide 6
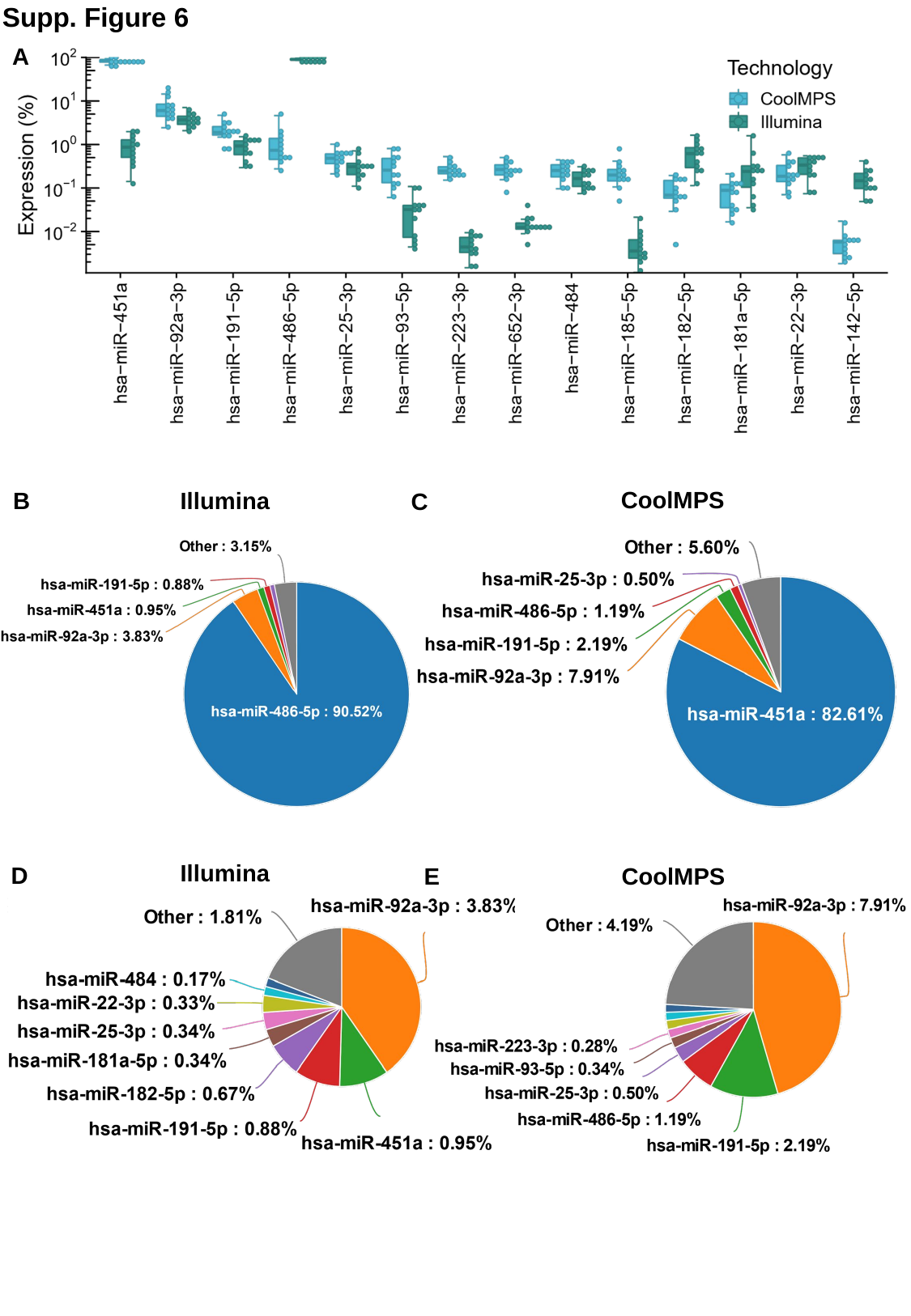

Supp. Figure 6
A
CoolMPS
Illumina
B
C
Illumina
D
CoolMPS
E
