# Supplementary material for: CoolMPS: evaluation of antibody labeling based massively parallel non-coding RNA sequencing
Source: Nucleic Acids Res. 2020 Dec 8;49(2):e10. doi: 10.1093/nar/gkaa1122 (PMC7826284; doi:10.1093/nar/gkaa1122)
Supplement: gkaa1122_Supplemental_Files [file gkaa1122_supplemental_files.zip › Supplemental Legends.docx]

**Supplemental Figure Legends**

**Supplemental Figure 1. Quality control metrics. A.**Distribution of the overall GC content of the unprocessed samples. The vertical lines delimit the quantiles.**B.**Distribution of the overall GC content of the processed samples after adapter and quality trimming. The vertical lines delimit the quantiles. **C.**Mean quality scores over all unprocessed samples per technology as function of the position in the read. The smoothed curve is fitted by a loess regression model. The grey area represents the confidence interval of the fit. **D.**Mean quality scores over all processed samples after adapter and quality trimming per technology as function of the position in the read. The smoothed curve is fitted by a loess regression model. The grey area represents the confidence interval of the fit. **E.** Distribution of the estimated error rate of the processed samples. Each sample is shown as one dot. The boxes span the first to the third quartile with the horizontal line inside the box representing the median value. The whiskers show the minimum and maximum values or values up to 1.5 times the interquartile range below or above the first or third quartile if outliers are present.  **F.** Distribution of the average per read GC content of the unprocessed samples. The gray area represents the standard deviation. **G.**Distribution of the average per read GC content of the processed samples after adapter and quality trimming. The gray area represents the standard deviation. **H.** Distribution of the read length after adapter and quality trimming. Reads shorter than 17 nt were discarded. The smoothed curve is fitted by a loess regression model. The grey area represents the confidence interval of the fit. **I.** Distribution of the average read duplication levels of the unprocessed samples. The gray area represents the standard deviation. **J.** Distribution of the average read duplication levels of the processed samples after adapter and quality trimming samples. The gray area represents the standard deviation. **K.** Average base composition of the unprocessed samples. The gray area represents the standard deviation. **L.** Average base composition of the processed samples after adapter and quality trimming. The gray area represents the standard deviation.

**Supplemental Figure 2. Distribution of overrepresented CoolMPS miRNAs in technical replicates.**Distribution of the average miRNA expression, in the subsampled BGISEQ and CoolMPS technical replicates, shown as boxplot (left) and dotplot (right). Each sample is shown as one dot. The boxes span the first to the third quartile with the horizontal line inside the box representing the median value. The whiskers show the minimum and maximum values or values up to 1.5 times the interquartile range below or above the first or third quartile if outliers are present.

**Supplemental Figure 3. Predicted miRNA candidates. A.**Venn diagram showing the overlap of the miRNA candidates predicted using the CoolMPS samples and the BGISEQ samples. **B.**NovoMiRank score distribution of the CoolMPS and BGISEQ specific miRNA candidates, as well as the candidates predicted in both technologies. The vertical lines delimit the quantiles.

**Supplemental Figure 4. CoolMPS vs Illumina quality control. A.**Distribution of the average Q30 value per sample for the two technologies, shown as boxplot (left) and dotplot (right). Each sample is shown as one dot. The boxes span the first to the third quartile with the horizontal line inside the box representing the median value. The whiskers show the minimum and maximum values or values up to 1.5 times the interquartile range below or above the first or third quartile if outliers are present.**B.**Q30 value over all samples per technology as function of the position in the read. The smoothed curve is fitted by a generalized additive model using a cubic regression spline. The grey area represents the confidence interval of the fit. **C.** Distribution of the percentage of reads mapping to the human reference genome hg38 without mismatch per technology, shown as boxplot (left) and dotplot (right). Each sample is shown as one dot. The boxes span the first to the third quartile with the horizontal line inside the box representing the median value. The whiskers show the minimum and maximum values or values up to 1.5 times the interquartile range below or above the first or third quartile if outliers are present. **D.**Scatter plot of the average expression of all miRNAs and all samples for the two technologies. The blue line is the regression line. The Pearson correlation is shown in the upper left part of the plot. The ten miRNAs with the largest fold change between both technologies are highlighted. **E.**Heat map of the clustered expression z-scores of miRNAs (rows) and samples (columns). The color code for the columns represents the technology. The dendrogram shows the hierarchical clustering of the samples with Euclidean distance and complete linkage. **F.**Distribution of all 24*23/2=276 pair wise Pearson correlation coefficients, shown as violin plot. The black box spans the first to the third quartile and the white dot shows the median. **G.**Correlation matrix of the expression values of all miRNAs for all samples. The dendrogram shows the hierarchical clustering of the samples with Euclidean distance and complete linkage.

**Supplemental Figure 5. Distribution to the different sncRNAs classes for CoolMPS and Illumina samples. A.**Donut plot comparing the distribution of all RNA classes and intergenic regions that were covered by reads from CoolMPS and Illumina.**B.**Scatter plot that shows the percentage of reads mapping to the RNA classes and intergenic regions for Illumina (x-axis) and CoolMPS (y-axis) on a logarithmic scale.

**Supplemental Figure 6. Distribution to microRNAs for CoolMPS and Illumina samples. A.**Distribution of the percentage of the 10 most abundant miRNAs in the CoolMPS and Illumina data, shown as boxplot (left) and dotplot (right). Each sample is shown as one dot. The boxes span the first to the third quartile with the horizontal line inside the box representing the median value. The whiskers show the minimum and maximum values or values up to 1.5 times the interquartile range below or above the first or third quartile if outliers are present. **B**. Pie chart for the top five miRNAs in the Illumina samples. **C.** Pie chart for the top five miRNAs in the CoolMPS samples.**D.**Pie chart for the top ten miRNAs in the Illumina samples after exclusion of the most abundant miR-486-5p. **E.**Pie chart for the top ten miRNAs in the CoolMPS samples after exclusion of the most abundant miR-451a.
